# Patient perceptions of glucocorticoid side effects: a cross-sectional survey of users in an online health community

**DOI:** 10.1136/bmjopen-2016-014603

**Published:** 2017-04-03

**Authors:** Ruth Costello, Rikesh Patel, Jennifer Humphreys, John McBeth, William G Dixon

**Affiliations:** 1 Arthritis Research UK Centre for Epidemiology, Centre for Musculoskeletal Research, School of Biological Sciences, Manchester Academic Health Science Centre, The University of Manchester, Manchester, UK; 2 NIHR Manchester Musculoskeletal Biomedical Research Unit, Central Manchester University Hospitals NHS Foundation Trust, Manchester Academic Health Science Centre, Manchester, UK; 3 Health eResearch Centre, Manchester Academic Health Science Centre, The University of Manchester, Manchester, UK

**Keywords:** Steroids, Side effects, Patient beliefs

## Abstract

**Objectives:**

To identify the side effects most important to glucocorticoid (GC) users through a survey of a UK online health community (Healthunlocked.com).

**Design:**

Online cross-sectional survey.

**Setting:**

Participants were recruited through Healthunlocked.com, an online social network for health.

**Participants:**

Adults who were currently taking GCs, or had taken GCs in the past month.

**Method:**

Responders scored the importance of listed side effects from 1 to 10, with 10 being of high importance to them. For each side effect, histograms were plotted, and the median rating and IQR were determined. Side effects were ranked by median ranking (largest to smallest) and then IQR (smallest to largest). The scores were categorised as low (scores 1–3), medium (scores 4–7) and high (scores 8–10) importance.

**Results:**

604 responders completed the survey. Histograms of side effect scores showed a skew towards high importance for weight gain, a U-shaped distribution for cardiovascular disease (CVD), diabetes, eye disease and infections, and a skew towards low importance for acne. When ranked, the side effect of most importance to responders was weight gain (median score=9, IQR 6–10) followed by insomnia and moon face with equal median score (8) and IQR (5–10). Three serious side effects, CVD, diabetes and infections, were ranked of lower importance overall but had wide ranging scores (median score=8, IQR 1–10).

**Conclusions:**

The three most highly rated side effects were not clinically serious but remained important to patients, perhaps reflecting their impact on quality of life and high prevalence. This should be taken into consideration when discussing treatment options and planning future GC safety studies.

**Strengths and limitations of this study**

- This survey used a novel recruitment method, through an online social network for health, which resulted in over 600 UK respondents who were taking glucocorticoids for a variety of conditions.
- Only a few studies have previously investigated which glucocorticoid side effects are most important to patients.
- The sample was mainly female and over 50 years of age, which may represent bias in the type of people who participate in studies.

## Introduction

Glucocorticoids (GC) continue to be widely used to treat inflammatory diseases since their discovery over 60 years ago.^1^ In the UK, around 1% of the population have been prescribed oral GCs, most commonly in the context of respiratory disease.^2^ For certain conditions, such as vasculitis, systemic lupus erythematosus and polymyalgia rheumatica, GCs are used in nearly all patients.^3^ ^4^

GCs have many side effects, ranging from potentially life-threatening such as cardiovascular events and infections,^5–7^ to less clinically serious effects such as bruising, skin thinning and fat redistribution. Understandably, research to date has focused more on the serious side effects, but these ‘less serious’ side effects may be important to the patient and have the potential to markedly impair a patient's quality of life. Furthermore, patients may elect not to take GC therapy because of concerns about possible side effects. To date, only a few studies have investigated which side effects are important to patients.^8–10^ Although osteoporosis was in the top three most important side effects in two of the three studies, the findings in general have not been consistent. For example, in one study ‘diabetes/glucose intolerance’ was ranked third most important,^8^ while in another ‘trouble with blood glucose levels/diabetes’ was 12th of side effects that bothered patients a lot.^10^ Two of these studies were in patients with specific diseases, adrenal insufficiency (where GCs are used to replace deficient endogenous GCs)^9^ and immune thrombocytopenic purpura (ITP),^10^ while the third studied patients with rheumatic diseases.^8^ Observed differences between these studies might be explained by the use of GC therapy for the treatment of inflammatory disease versus replacement therapy. To understand which side effects are most important to patients across several disease groups, the aim of this study was to identify the side effects most important to GC users through a survey of multiple disease communities within a UK online health social media platform (Healthunlocked.com (HU)).

## Methods

### Setting

HU is a social network for health where patients, caregivers and health advocates can discuss issues related to their health through online message boards and private messages. Discussions take place within communities set up by patient charities and condition communities from NHS Choices. It is the largest health-related social network in Europe with 4 million visitors per month. The HU platform allows rapid access to hundreds of potential GC users by embedding a survey in posts with a particular title word, or tagged with a given word or phrase.

### Design

A short survey about GC use, timing of GC administration and perceptions of side effects was designed by the research team specifically for this study. The survey was drafted by WD to include information about people's beliefs about the importance of range of known serious and non-serious GC-associated side effects. The number of items was selected to balance the burden of data entry with collecting opinion on a range of side effects, including items scored of high, intermediate and low importance in previous studies.^8–10^ The draft was further refined with input from rheumatologists, endocrinologists and epidemiologists (RP, JH, JMcB, RC plus wider consultation with local colleagues (see acknowledgements)). This resulted in some rewording of questions and two additional side effects were added: ‘changes in mood’ and ‘round face or “moon” face’. The survey was then piloted with 13 members of an existing musculoskeletal Research User Group (RUG), comprised of patients with musculoskeletal disease and their carers who meet quarterly to help support research studies. RUG members were asked to comment on comprehension, ease of completion and provide any general feedback. The survey was finalised based on the pilot testing responses. No additional GC-associated adverse events were suggested for inclusion in the survey by the patient group (see online supplementary material S1). The testing supported our decision to ask participants to score rather than rank each item. One reviewer commented, “I always find it hard to do the thing where they ask you to rank items—in this case, rank this list of side effects from highest to lowest (importance to you) and so I think the system you have used is better. And anyway, a heart attack is surely never going to anywhere other than at the top of the list of undesirable outcomes.”

10.1136/bmjopen-2016-014603.supp1 supplementary material

The survey popped up on HU posts that included either the title word ‘steroid’ or the tags ‘glucocorticoid’, ‘prednisolone’, ‘prednisone’, ‘steroid’ or ‘dexamethasone’ and was restricted to UK users. When the survey popped up, the community group for the post was recorded automatically for each responder. To avoid recall bias, only respondents who were currently using, or had used GCs in the last month were eligible. To determine eligibility, a stem question asked whether the respondent was currently taking oral steroids, or had taken oral steroids within the last month. If the response was ‘No’, the survey ended. If the response was ‘Yes’, the survey continued. The survey started in December 2015 was live for 3 months or until 1000 surveys were completed, whichever came first. No formal sample size was calculated. Recruitment targets were instead based on discussions with HU about anticipated response rates over a 3-month period.

The perception of GC side effects was examined by asking respondents, ‘Please score each side effect, even if you have not experienced it, on a scale where 1= very little importance and 10= high importance to you’. Side effects were listed alphabetically as follows: acne, cardiovascular disease (eg, heart attack), changes in mood, diabetes, eye disease (cataracts, glaucoma), high blood pressure, indigestion, infection (eg, pneumonia), insomnia (unable to sleep), palpitations (racing heart), reduced bone strength (osteoporosis, fractures), round face or ‘moon’ face, skin changes (bruising, thin skin, stretch marks) and weight gain. Experience of side effects was examined by asking respondents, ‘Have you had any of these side effects whilst taking steroids?’ Respondents could indicate any that applied.

### Statistical analysis

The scores for each side effect were plotted on histograms, and the median score and IQR was determined. Side effects were ranked by median score (largest to smallest) and then IQR (smallest to largest) for those with the same median, to identify the most important side effects to patients. The scores for each side effect were categorised as low importance (scores 1–3), medium importance (scores 4–7) and high importance (scores 8–10). Side effect scores were then stratified by community group and experience of side effects. Median side effect scores and IQR, stratified by experience, were displayed in a box and whisker plot. Respondents with missing data for side effect scores were not included in the analysis.

## Results

### Patient characteristics

The survey was live for 3 months, it popped up for 17 999 visitors, and 1311 (7.1%) clicked on the survey. Of those, 756 (58%) agreed to take part in the survey, 664 (51%) were eligible and 604 (46%) provided complete data (see online supplementary figure S1).

10.1136/bmjopen-2016-014603.supp2 supplementary figure Flowchart of survey respondents.

Patients came from five community groups: British Lung Foundation (BLF) (N=54), ITP support (N=17), Lupus UK (N=82), National Rheumatoid Arthritis Society (NRAS) (N=229) and Polymyalgia Rheumatica and Giant Cell Arteritis UK (PMRGCAUK) (N=221). The majority of completers were over 50 years old (81%) and women (86%). Those who dropped out part way through the survey (n=60) were not significantly different from those who completed the survey in terms of age, gender and community (table 1).

**Table 1:** Characteristics of survey responders who completed the survey and those who dropped out during the survey (N=664)

	Completed survey (n=604) N (%)	Dropped out during survey (n=60) N (%)
Community group		
BLF	54 (8.9)	10 (16.7)
ITP support	17 (2.8)	1 (1.7)
Lupus UK	82 (13.6)	6 (10)
NRAS	229 (37.9)	22 (36.7)
PMRGCAUK	221 (36.6)	19 (31.7)
Missing	1 (0.2)	2 (3.3)
Age (years)		
Under 39	40 (6.6)	4 (6.7)
40–49	77 (12.7)	5 (8.3)
50–59	201 (33.3)	14 (23.3)
60–69	181 (30)	19 (31.7)
70 years or over	105 (17.4)	16 (26.7)
Missing	0 (0)	2 (3.3)
Gender		
Male	79 (13.1)	6 (10)
Female	522 (86.4)	49 (81·7)
Missing	3 (0.5)	5 (8·3)
Total	604	60

BLF, British Lung Foundation; ITP, immune thrombocytopenia; NRAS, National Rheumatoid Arthritis Society; PMRGCAUK, Polymyalgia Rheumatica and Giant Cell Arteritis UK.

### Survey responses

**Figure 1:**
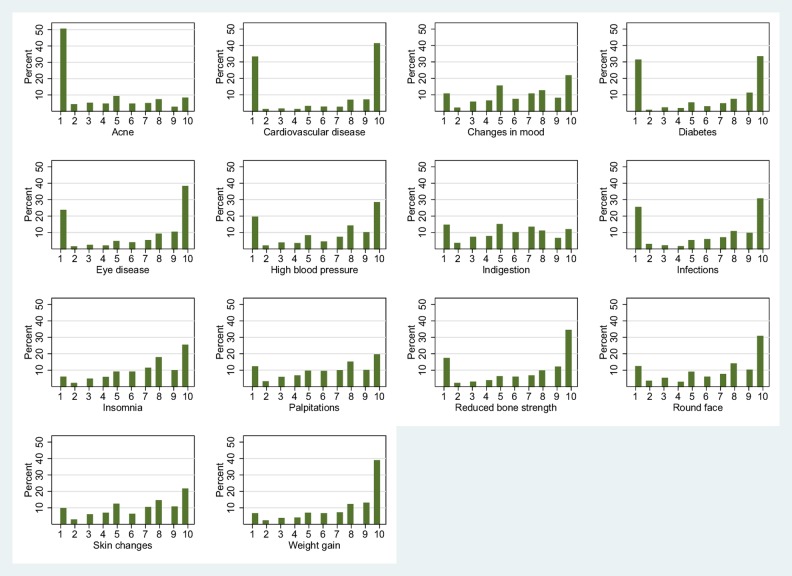
Histograms of side effect ratings. 1= rating of lowest importance, 10= rating of highest importance.

Figure 1 shows histograms of scores for each side effect. Comparing across histograms, weight gain scores show a pronounced skew towards high importance. Cardiovascular disease (CVD), diabetes, eye disease and infections scores have a U-shaped distribution of scores. Acne scores show a pronounced skew towards low importance.

When ranked, weight gain was the side effect of most importance (median score =9, IQR 6–10), with 64% of weight gain scores categorised as high importance. Insomnia, moon face, high blood pressure (BP), reduced bone strength, eye disease, CVD, diabetes and infection all had the same median score of 8; however, the range of scores varied. Insomnia and moon face were ranked joint second as they had the smallest range of scores (IQR 5–10). Insomnia, like weight gain, had only 12% of respondents who rated it as low importance, whereas all other side effects with a median of 8 were rated as low importance by at least 20% of participants. Side effects with a median score below 8 had <50% of scores categorised as high importance (table 2).

**Table 2:** Median, IQR, rank and categories of side effect scores

Symptom	Median (IQR)	Rank	Low (score 1–3) N (%)	Medium (score 4–7) N (%)	High (score 8–10) N (%)
Weight gain	9 (6–10)	1	74 (12.3)	145 (24)	385 (63.7)
Insomnia	8 (5–10)	2	75 (12.4)	210 (34.8)	319 (52.8)
Moon face	8 (5–10)	2	125 (20.7)	149 (24.7)	330 (54.6)
High blood pressure	8 (4–10)	4	150 (24.8)	138 (22.8)	316 (52.3)
Reduced bone strength	8 (4–10)	4	133 (22)	134 (22.2)	337 (55.8)
Eye disease	8 (3–10)	6	164 (27.2)	93 (15.4)	347 (57.5)
Cardiovascular disease	8 (1–10)	7	216 (35.8)	56 (9.3)	332 (55)
Diabetes	8 (1–10)	7	206 (34.1)	86 (14.2)	312 (51.7)
Infections	8 (1–10)	7	182 (30.1)	116 (19.2)	306 (50.7)
Changes in mood	7 (5–9)	10	110 (18.2)	239 (39.6)	255 (42.2)
Skin changes	7 (5–9)	10	109 (18)	214 (35.4)	281 (46.5)
Palpitations	7 (4–9)	12	125 (20.7)	212 (35.1)	267 (44.2)
Indigestion	6 (3–8)	13	152 (25.2)	276 (45.7)	176 (29.1)
Acne	1 (1–6)	14	359 (59.4)	138 (22.8)	107 (17.7)

When stratified by community group the rankings remained similar to the overall rankings for all communities except the PMRGCAUK community group, where the side effects most important to respondents were eye disease, CVD and insomnia, with weight gain fourth (table 3).

**Table 3:** Median, IQR and rank of side effect scores, stratified by community group

	BLF (N=54)		ITP support (N=17)		Lupus UK (N=82)		NRAS (N=229)		PMRGCAUK (N=221)	
Symptom	Median (IQR)	Rank	Median (IQR)	Rank	Median (IQR)	Rank	Median (IQR)	Rank	Median (IQR)	Rank
Acne	1 (1–5)	14	2 (1–7)	14	3 (1–6)	14	1 (1–6)	14	1 (1–6)	13
Cardiovascular disease	6.5 (1–10)	11	5 (1–10)	12	8 (1–10)	8	8 (1–10)	7	9 (1–10)	2
Changes in mood	7 (4–10)	9	6 (5–8)	8	8 (4–10)	6	7 (4–9)	10	7 (5–9)	9
Diabetes	7 (1–10)	10	4 (1–9)	13	7 (1–9)	12	8 (1–10)	7	8 (1–10)	8
Eye disease	7.5 (1–10)	6	8 (1–9)	5	8 (3–10)	7	8 (3–10)	4	9 (4–10)	1
High blood pressure	7.5 (4–10)	5	6 (4–8)	10	8 (5–9)	4	8 (3–10)	4	8 (3–10)	7
Indigestion	6 (4–8)	12	7 (5–8)	6	6 (3–8)	13	6 (4–8)	13	6 (3–8)	13
Infections	8.5 (3–10)	2	5 (1–9)	11	8 (5–10)	5	8 (2–10)	6	7 (1–10)	12
Insomnia	8 (7–10)	3	8 (7–10)	2	7 (5–9)	10	7 (5–9)	9	8 (6–10)	3
Palpitations	7 (4–9)	7	6 (4–7)	8	7.5 (5–9)	9	7 (4–9)	10	7 (4–9)	11
Reduced bone strength	8 (5–10)	4	8 (7–10)	2	9 (5–10)	2	8 (4–10)	3	8 (4–10)	6
Round face	6 (3–10)	13	8 (5–8)	2	8.5 (5–10)	3	8 (5–10)	2	8 (5–10)	5
Skin changes	7 (3–8)	7	7 (3–8)	7	7 (4–9)	11	7 (5–10)	10	7 (5–9)	9
Weight gain	9 (5–10)	1	9 (8–9)	1	9 (7–10)	1	9 (6–10)	1	8 (6–10)	3

BLF, British Lung Foundation; ITP, immune thrombocytopenia; NRAS, National Rheumatoid Arthritis Society; PMRGCAUK, Polymyalgia Rheumatica and Giant Cell Arteritis UK.

When stratified by prior experience, participants who had previously experienced the side effect of interest reported higher median scores, with smaller IQRs. The side effects most important to those who had experienced them were diabetes, eye disease and CVD, all scoring a median of 10. The side effects most important to those who had not experienced them were reduced bone strength, CVD and eye disease (table 4, see online supplementary figure S2). Although weight gain had the highest rank overall, it was ranked only fourth in those who had and eighth in those who had not experienced it prior to completing the survey, with median scores and IQRs of 9 (7–10) and 6 (2–9), respectively. The most commonly experienced side effects were, in order, weight gain, round face, insomnia, changes in mood, skin changes and indigestion, all of which were experienced by over half of the 604 respondents.

**Table 4:** Median, IQR and rank of side effect scores, stratified by experience of side effect

	Experienced side effect			Did not experience side effect		
Symptom	N	Median (IQR)	Rank	N	Median (IQR)	Rank
Acne	47	6 (5–8)	14	557	1 (1–5)	14
Cardiovascular disease	29	10 (6–10)	3	575	8 (1–10)	2
Changes in mood	356	8 (5–10)	10	248	5 (2–8)	11
Diabetes	66	10 (8–10)	1	538	7 (1–10)	4
Eye disease	117	10 (7–10)	2	487	8 (1–10)	2
High blood pressure	203	9 (7–10)	4	401	6 (1–9)	9
Indigestion	304	7 (5–9)	13	300	5 (1–7)	11
Infections	133	8 (6–10)	9	471	7 (1–10)	5
Insomnia	381	8 (7–10)	8	223	6 (4–8)	6
Palpitations	259	8 (5–10)	10	345	6 (3–8)	7
Reduced bone strength	162	9 (6–10)	7	442	8 (3–10)	1
Round face	383	9 (7–10)	4	221	5 (1–8)	13
Skin changes	348	8 (5–10)	10	256	5 (3–8)	10
Weight gain	442	9 (7–10)	4	162	6 (2–9)	8

10.1136/bmjopen-2016-014603.supp3 supplementary figure Box and whisker plot of side effect scores, by whether the side effects were experienced. Horizontal bars represent the median and the vertical lines represent the inter-quartile range.

## Discussion

It is known that oral GCs have many side effects, but few studies have investigated which matter the most to patients. This survey found that overall weight gain, insomnia and moon face were the side effects ranked highest by patients, despite them being less clinically serious. The importance of side effects to respondents was different depending on whether they had been experienced, with clinically serious side effects (diabetes, eye disease and CVD) being most important to respondents who had experienced them. As these clinically serious side effects had not been experienced by the majority of respondents, they dropped in the rankings overall. Weight gain, scored at 9 out of 10 for those who had experienced it and 6 out of 10 for those who had not, ranking at fourth position in both groups, but rose to the top ranking overall because of its high prevalence having been experienced by 442/604 (73%) participants. Participants from the PMRGCAUK community rated eye disease as most important, with CVD second and insomnia and weight gain joint third. This contrasted to all other communities where weight gain was the most important side effect overall. This group may be taking a higher dose of GC, compared with the other communities, which may explain the difference. Alternatively, respondents from this community may be older, and thus could be more concerned about diseases more prevalent at this higher age. Awareness of potential ocular involvement of giant cell arteritis (GCA) may also make the possible occurrence of further eye disease particularly concerning.

Clinicians and patients make treatment decisions after weighing the benefits against the possible harms, and for each benefit or harm, considering its probability, its nature, and a value judgement of how important it is to the individual.^11^ While many studies have estimated the frequency of side effects, few have considered how important they are to patients.^12–15^ This is relevant because patients' value judgements about a given side effect will influence their decisions about treatment and adherence.^13^ ^16^ Three prior studies have investigated patient perspectives of GC side effects specifically. The most cited of these is a study comparing the perspectives of 140 patients and 110 rheumatologists. They found osteoporosis was ‘the most worrisome’ side effect for patients, followed by CVD, diabetes, weight gain and renal dysfunction.^8^ The other two studies were interested in specific disease groups. One study of patients with ITP found the most bothersome side effects of those experienced, in line with our findings, were moon face, weight gain and insomnia.^10^ Another study of patients with adrenal insufficiency found the most worrisome side effect was osteoporosis, followed by obesity and fatigue.^9^ In all studies, weight gain was one of the top five most worrisome side effects, which is in agreement with our findings. Weight gain is known to adversely affect body image and self-esteem, although there are no studies, to the best of our knowledge, examining the impact of GC-associated weight gain on quality of life. A few studies have reported on weight gain following GC therapy.^14^ ^17^ ^18^ However, studies are often not designed to measure this as an outcome and as a result, fail to address the sort of questions that patients may be interested in, such as the extent of weight gain with specific doses, or the likelihood of weight loss following discontinuation. Despite the importance to patients of insomnia in this and other studies, it is interesting to note that very few studies have investigated insomnia in patients taking GC therapy.

This study used a novel method of recruiting survey respondents, through a social networking website, which was easy to conduct and resulted in a sample of just over 600 UK-based respondents who were taking GCs for a variety of conditions. However, there were limitations of the study. The sample was mainly female and over 50 years of age: this may be partly due to the disease demographic, but may also represent a selection bias in the types of people who are more likely to participate in studies,^19^ or participate in a social network. This selection factor may have influenced our findings if perceptions of the importance of side effects could be different between the sexes. For example, female participants may be more inclined to see weight gain as important. It may also affect the generalisability of the results, as the scores may not represent the views of the whole population, for example, young men are not well represented. It relied on self-report to identify steroid users. However, a previous study showed high agreement between self-reported medication use and pharmacy records, so it is unlikely there will be large misclassification due to self-report.^20^ We did not collect information about comorbidities in participants and were thus unable to examine how this may have influenced beliefs. For example, a patient with prevalent hypertension may have considered high BP or CVD to be particularly important to them as a GC-associated side effect. Nonetheless, our results reflect the patients' experiences and how they rate the importance of serious and non-serious outcomes. It was particularly interesting to note the distribution of responses in the high-ranking serious and non-serious conditions. For weight gain and insomnia, only 12% of participants scored them as low importance. Yet although CVD and diabetes had a median score of 8 (like insomnia), there was a U-shaped distribution of scores where more than 20% of participants scored them as low importance despite their seriousness. It may be that education influenced scores: if respondents were not aware of the risks of CVD with GCs, for example, they may not have scored CVD as important to them. Unfortunately, we did not collect information on education. Another explanation may be that some respondents may have had optimism bias,^21^ where respondents believed that the serious side effects would not happen to them. This could also result in the wide variation of scores for serious side effects.

In conclusion, this study has shown that weight gain, insomnia and moon face were the top three most important side effects to patients taking GCs. Despite this, they are not widely studied with many unanswered questions. Research should be informed by patients, and targeted to provide patients with better information about these side effects of high importance.
