# Supplementary material for: Patient perceptions of glucocorticoid side effects: a cross-sectional survey of users in an online health community
Source: BMJ Open. 2017 Apr 3;7(4):e014603. doi: 10.1136/bmjopen-2016-014603 (PMC5387953; doi:10.1136/bmjopen-2016-014603)
Supplement: supplementary material [file bmjopen-2016-014603supp.pdf]

Timing and outcomes of steroid use

We'd like to invite you to take this short survey, which aims to investigate the time at which patients take their steroids tablets (E.g. prednisolone, betamethasone, deflazacort, calcort, dexamethasone, hydrocortisone, methylprednisolone and medrone) and what side effects patients view as being important.

Before you decide whether you want to take part, it is important for you to understand why the survey is being done and what your participation will involve. Please take time to read the following information carefully:

● The survey is being funded by the Medical Research Council and conducted by the University of Manchester, in partnership with HealthUnlocked.
● All information you provide will be anonymous and treated in the strictest confidence and according to legal and ethical guidelines of the UK Data Protection Act 1998.
● The information you provide will be stored at the University of Manchester for 10 years.
● If you decide to take part you are still free to withdraw at any time during the survey. However, as data collection is anonymous and sent when the survey is completed, your information cannot be identified to withdraw after survey completion.

The total completion of the survey should not take more than 2 minutes

☐ **Tick this box if you understand the above information and agree to take part**

Do you take <u>oral steroid tablets</u> (or have taken them within the last month)?

Examples of steroid tablets include prednisolone, betamethasone, deflazacort, calcort, dexamethasone, hydrocortisone, methylprednisolone and medrone.

- ◯ Yes
- ◯ No

1. How old are you?

- under 19
- 20 - 29
- 30 - 39
- 40 - 49
- 50 - 59
- 60 - 69
- 70 or more

2. Which gender best describes you?

- ◯ Male
- ◯ Female

3. Do you take your daily dose of steroids once per day, or do you split the dose over two or more times through the day?

- ◯ Once per day
- ◯ Two or more

4. What time do you normally take your steroid tablets?

Please enter the nearest time when taken most often

	hh		mm	AM/PM
Time		:		-

4. What time do you normally take your steroid tablets?

Please enter the nearest time when taken most often.

First dose:

	hh		mm	AM/PM
Time		:		-

Second dose:

	hh		mm	AM/PM
Time		:		-

Third dose:

	hh		mm	AM/PM
Time		:		-

5. We are interested to learn how important a range of possible side effects is to you.

Please score each side effect, <u>even if you have not experienced it</u>, on a scale where 1=very little importance, and 10=high importance to you.

	1	2	3	4	5	6	7	8	9	10
Acne	○	○	○	○	○	○	○	○	○	○
Cardiovascular disease (e.g. heart attack)	○	○	○	○	○	○	○	○	○	○
Changes in mood	○	○	○	○	○	○	○	○	○	○
Diabetes	○	○	○	○	○	○	○	○	○	○
Eye disease (cataracts, glaucoma)	○	○	○	○	○	○	○	○	○	○
High blood pressure	○	○	○	○	○	○	○	○	○	○
Indigestion	○	○	○	○	○	○	○	○	○	○
Infection (e.g. pneumonia)	○	○	○	○	○	○	○	○	○	○
Insomnia (unable to get to sleep)	○	○	○	○	○	○	○	○	○	○
Palpitations (racing heart)	○	○	○	○	○	○	○	○	○	○
Reduced bone strength (osteoporosis, fractures)	○	○	○	○	○	○	○	○	○	○
Round face, or ‘moon’ face	○	○	○	○	○	○	○	○	○	○
Skin changes (bruising, thin skin, stretch marks)	○	○	○	○	○	○	○	○	○	○
Weight gain	○	○	○	○	○	○	○	○	○	○

Other (please specify)

[ ]

6. Have you had any of these side effects whilst taking steroids?

Please select all that apply

- [ ] Acne
- [ ] Cardiovascular disease (e.g. heart attack)
- [ ] Changes in mood
- [ ] Diabetes
- [ ] Eye disease (cataracts, glaucoma)
- [ ] High blood pressure
- [ ] Indigestion
- [ ] Infection (e.g. pneumonia)
- [ ] Insomnia (unable to get to sleep)
- [ ] Palpitations (racing heart)
- [ ] Reduced bone strength (osteoporosis, fractures)
- [ ] Round face, or ‘moon’ face
- [ ] Skin changes (bruising, thin skin, stretch marks)
- [ ] Weight gain
- [ ] None of the above / Other (please specify)
