# Supplementary figures and images for: Patient perceptions of glucocorticoid side effects: a cross-sectional survey of users in an online health community
Source: BMJ Open. 2017 Apr 3;7(4):e014603. doi: 10.1136/bmjopen-2016-014603 (PMC5387953; doi:10.1136/bmjopen-2016-014603)

Figure S1: Flowchart of survey respondents.

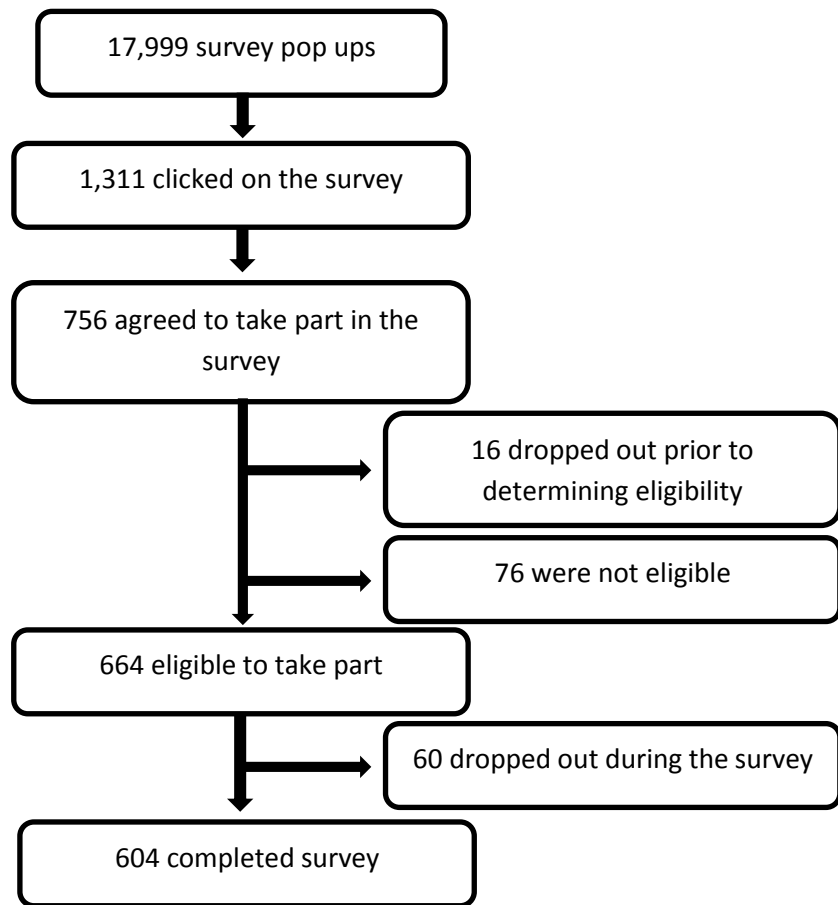

Supplement: supplementary figure — Flowchart of survey respondents. [file bmjopen-2016-014603supp_figure1.pdf]
