# Supplementary material for: Patient perceptions of glucocorticoid side effects: a cross-sectional survey of users in an online health community
Source: BMJ Open. 2017 Apr 3;7(4):e014603. doi: 10.1136/bmjopen-2016-014603 (PMC5387953; doi:10.1136/bmjopen-2016-014603)

Figure S2: Box and whisker plot of side effect scores, by whether the side effects were experienced. Horizontal bars represent the median and the vertical lines represent the inter-quartile range.

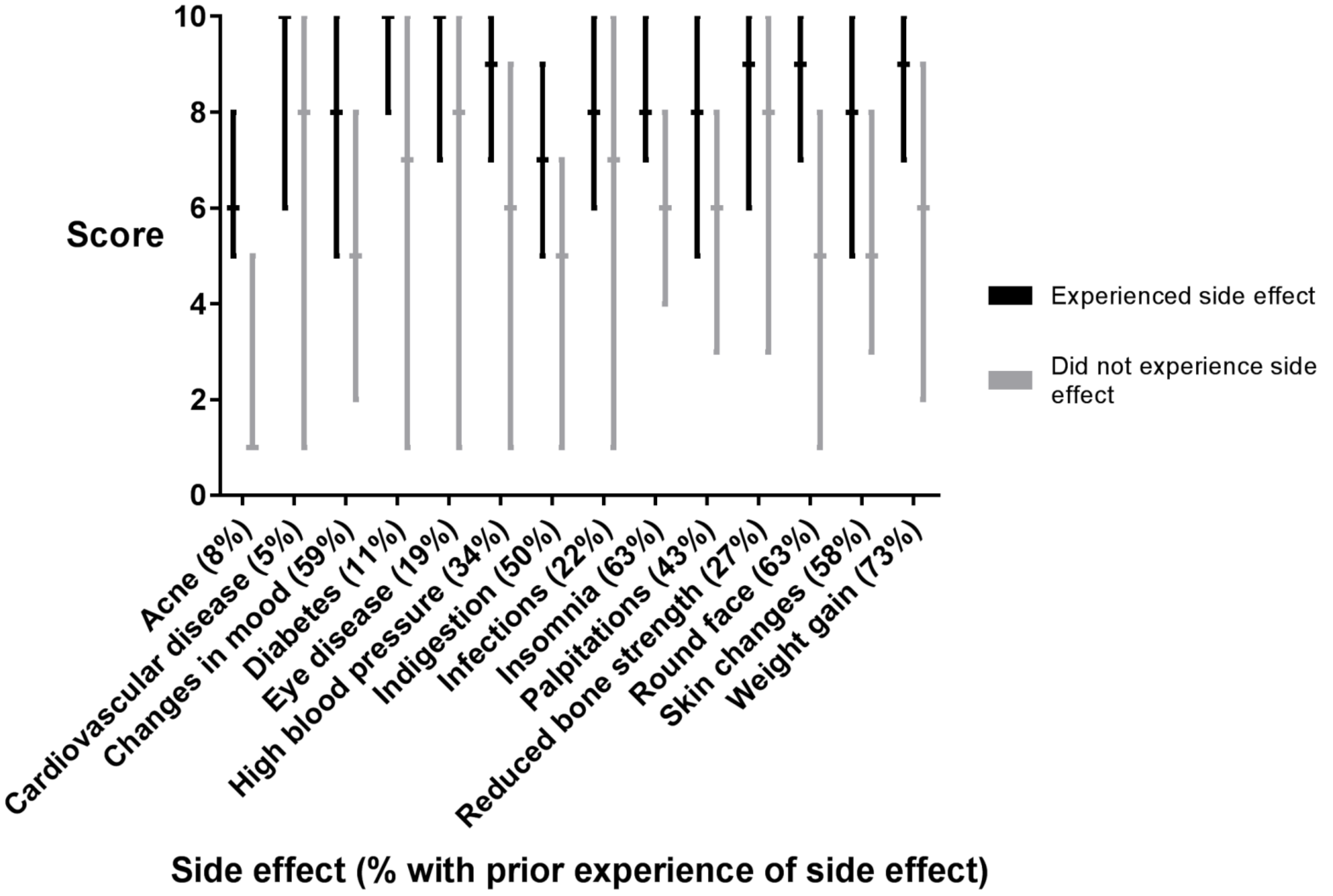

Supplement: supplementary figure — Box and whisker plot of side effect scores, by whether the side effects were experienced. Horizontal bars represent the median and the vertical lines represent the inter-quartile range. [file bmjopen-2016-014603supp_figure2.pdf]
